# Role of Trehalose in Salinity and Temperature Tolerance in the Model Halophilic Bacterium *Chromohalobacter salexigens*

**DOI:** 10.1371/journal.pone.0033587

**Published:** 2012-03-20

**Authors:** Mercedes Reina-Bueno, Montserrat Argandoña, Manuel Salvador, Javier Rodríguez-Moya, Fernando Iglesias-Guerra, Laszlo N. Csonka, Joaquín J. Nieto, Carmen Vargas

**Affiliations:** 1 Department of Microbiology and Parasitology, University of Seville, Seville, Spain; 2 Department of Organic and Pharmaceutical Chemistry, University of Seville, Seville, Spain; 3 Department of Biological Sciences, Purdue University, West Lafayette, Indiana, United States of America; Universidad Miguel Hernandez, Spain

## Abstract

The disaccharide trehalose is considered as a universal stress molecule, protecting cells and biomolecules from injuries imposed by high osmolarity, heat, oxidation, desiccation and freezing. *Chromohalobacter salexigens* is a halophilic and extremely halotolerant γ-proteobacterium of the family *Halomonadaceae*. In this work, we have investigated the role of trehalose as a protectant against salinity, temperature and desiccation in *C. salexigens*. A mutant deficient in the trehalose-6-phosphate synthase gene (*otsA::*Ω) was not affected in its salt or heat tolerance, but double mutants ectoine- and trehalose-deficient, or hydroxyectoine-reduced and trehalose-deficient, displayed an osmo- and thermosensitive phenotype, respectively. This suggests a role of trehalose as a secondary solute involved in osmo- (at least at low salinity) and thermoprotection of *C. salexigens*. Interestingly, trehalose synthesis was osmoregulated at the transcriptional level, and thermoregulated at the post-transcriptional level, suggesting that *C. salexigens* cells need to be pre-conditioned by osmotic stress, in order to be able to quickly synthesize trehalose in response to heat stress. *C. salexigens* was more sensitive to desiccation than *E. coli* and desiccation tolerance was slightly improved when cells were grown at high temperature. Under these conditions, single mutants affected in the synthesis of trehalose or hydroxyectoine were more sensitive to desiccation than the wild-type strain. However, given the low survival rates of the wild type, the involvement of trehalose and hydroxyectoine in *C. salexigens* response to desiccation could not be firmly established.

## Introduction

Halophiles, which require NaCl for growth, and halotolerant species, which are able to cope with high NaCl concentrations live in habitats of high ionic strength, such as marine water and salt lakes, brines, salterns, saline soils, salted fish, meat, and other foods [1]. Most of them accumulate high intracellular concentrations of the so-called compatible solutes, in order to balance the osmotic pressure of the surrounding medium and maintain cell turgor pressure. Compatible solutes are low molecular-weight organic compounds, named so because they do not interact with macromolecules in detrimental ways [2], [3]. Although there is a diversity of compatible solutes, they fall into a few major chemical categories, such as sugars (i.e., sucrose, trehalose), polyols (i.e, sorbitol, mannitol), amino acids and derivatives (i.e. proline, glutamate, glutamine), betaines (i.e., glycine betaine) and ectoines (i.e. ectoine and hydroxyectoine) [3]. In microorganisms, the evolutionary selection for the accumulation of a specific compatible solute may not depend uniquely on its osmoprotective role, but also upon the additional benefits its accumulation provides such as increased tolerance to other environmental constraints frequently or sporadically present in the microorganism's niche [4]. Indeed, the role of compatible solutes goes beyond osmotic adjustment alone to protection of cells and cell components from freezing, desiccation, high temperature, and oxygen radicals, as well as to serve as sources of carbon, energy and nitrogen. Thus, it is very common for microorganisms to use a cocktail of compatible solutes, a strategy that allows the cell to adapt the compatible solute pool to different environmental injuries [3], [4], [5]. It is well known for bacterial systems (i.e, *E. coli* or *Halobacillus*) and archaea (methanogens) that there is a switch in osmolyte strategy responding to growth and environmental conditions. In addition, when one solute is no longer produced usually a second is used instead/or increased [3], [6], [7], [8]. Besides their role on bacterial stress adaptation, compatible solutes have important biotechnological applications as protectants of macromolecules, whole cells, tissues, and even organs [3], [4].

We have a fairly complete picture of the distribution of compatible solutes in most groups of halophilic bacteria and archaea, which in some cases is correlated with their position in the phylogenetic tree of life [9]. For instance, the capacity to synthesize ectoines seems to be more specific of halophilic/halotolerant *Proteobacteria, Actinobacteridae*, and *Firmicutes* [10], whereas trehalose or glutamate synthesis, or the accumulation of glycine betaine upon transport from the external medium or choline oxidation, are far more widespread in nature [3].

Trehalose (*O*-alpha,-D-glucosyl-[1→1]-alpha-D-glucoside) is found in archaea, bacteria, yeasts, fungi, plants and invertebrates. It is considered as a universal stress molecule, protecting cells and biomolecules from injuries imposed by high osmolarity, heat, oxidation, desiccation and freezing. In addition, trehalose is a source of carbon and energy, and a signaling molecule in specific metabolic pathways [11], [12], [13]. To date, five different enzymatic pathways have been described for trehalose synthesis [13], [14]. The most common and the best-studied route among different species involves the enzyme trehalose-6-phosphate synthase (OtsA), which catalyses the transfer of glucose from UDP-glucose to glucose-6-phosphate, leading to trehalose-6-phosphate. In a second step, trehalose-6-phosphate phosphatase (OtsB) catalyzes the hydrolysis of the phosphate group from the intermediate disaccharide to generate trehalose. This pathway has been extensively studied in many bacteria (i.e., *E. coli*), archaea, yeasts, insects and plants [11], [13], [15]. Trehalose may also be synthesized directly from maltose via trehalose synthase, which is encoded by *treS* [16]. The TreS pathway has been described in *Mycobacterium* sp., the thermophilic bacterium *Thermus thermophilus* and the radiation-resistant bacterium *Deinococcus radiodurans* [17]. A third pathway found in several bacteria [18], [19] and in the archaeon *Sulfolobus acidocaldarius* [20] converts the terminal unit of a glucose polymer to trehalose via maltooligosyl trehalose synthase, encoded by *treY*, and maltooligosyl trehalose trehalohydrolase, encoded by *treZ*. A trehalose phosphorylase (TreP) can also catalyze the synthesis of trehalose in the presence of glucose-1-phosphate and glucose in fungi. This catalytic reaction has only been shown *in vitro* [21]. Finally, a less common pathway involves the conversion of ADP-glucose and glucose, instead of glucose-6-phosphate, into trehalose. The enzyme responsible for this reaction, a trehalose glycosyl transferring synthase (TreT), has been characterized in the hyperthermophilic archaea *Termococcus litoralis* and *Pyrococcus horikoshii* [22], [23]. Most microorganisms have only a single pathway for the synthesis of trehalose (OtsA/OtsB), others have two, and a few even possess three or four pathways [18], [24], [25].

*Chromohalobacter salexigens* [26] is a halophilic gamma-proteobacterium of the family *Halomonadaceae*. It exhibits an extraordinary broad salt growth range: from 0.5 M to 3 M NaCl in minimal medium M63 [27], and from 0.15 to 4.3 M NaCl in complex saline medium SW-10 [23], being considered as a model microorganism to study prokaryotic osmoadaptation [26]. If available in the external medium, *C. salexigens* transports and accumulates glycine betaine, which can be also synthesized by oxidation of its precursor choline through the *betIBA*-encoded pathway [27], [28]. In addition, *C. salexigens* synthesizes *de novo* ectoines (ectoine and hydroxyectoine), and minor amounts of glutamate, glutamine, glucosylglycerate, and trehalose [29]. In the absence of osmoprotectants as glycine betaine, osmoadaptation is mainly achieved by the synthesis and accumulation of its major compatible solute, ectoine. Thus, one mutant affected in the synthesis of ectoine (strain CHR62, resulting from insertion of transposon Tn*1732* causing deletion of the ectoine synthesis genes *ectABC*), was unable to grow above 0.75 M NaCl in M63 medium [30]. In contrast, a mutant in which hydroxyectoine synthesis was reduced (resulting from inactivation of the main ectoine hydroxylase gene, *ectD*), was not osmosensitive, but thermosensitive, indicating that the main role of hydroxyectoine in *C. salexigens* was thermoprotection [31]. In agreement with the above findings, the accumulation of hydroxyectoine in *C. salexigens* is up-regulated by salinity and temperature, whereas the accumulation of ectoine is up-regulated by salinity and down-regulated by temperature [31]. In addition, ectoine and hydroxyectoine serve as carbon sources for *C. salexigens*, exclusively when grown under optimal growth conditions (1.5 M NaCl and 37°C) [32]. Whereas glutamate seems to accumulate in minor amounts in *C. salexigens* under all conditions tested, including growth with glycine betaine [33], glutamine has been only detected at 3 M NaCl [30]. Glucosylglycerate was first observed to accumulate in low amounts in a salt-sensitive mutant affected in the ectoine synthase gene [34] and latter on detected as a minor solute in *C. salexigens* wild type grown at low salinity and optimal temperature [33]. In addition, by using ^13^C-NMR, we showed that *C. salexigens* accumulates trehalose under high temperature conditions [31]. This observation was unexpected, as only traces of this solute can be detected in the wild-type strain grown at 37°C even at high salinity [30], [33]. Very interestingly, trehalose is also accumulated in high amounts at 37°C in the ectoine-deficient mutant strain CHR62 [30] grown at low salinity (0.75 M NaCl), suggesting that ectoine suppresses, either directly or indirectly, trehalose synthesis in the type strain under these conditions.

In this work, we investigated the role of trehalose in protection against three abiotic constraints (high salinity, high temperature, and desiccation stress) in *C. salexigens*. We analyzed genes for trehalose metabolism present in its genome and quantified the accumulation of trehalose and *otsA* gene expression in response to high salinity and high temperature. Our results suggest a role of trehalose as a secondary compatible solute involved in osmo- and thermoprotection of *C. salexigens*. However, the role of trehalose in desiccation tolerance could not be firmly demonstrated.

## Materials and Methods

### Bacterial strains, plasmids and culture conditions

Bacterial strains and plasmids used in this work are included in Table 1. All *C. salexigens* mutants were derived from strain CHR61 (a spontaneous Rf^r^ mutant of *C. salexigens* DSM 3043^T^; [26], [30]), and the latter strain was used as the control in all experiments. *C. salexigens* strains were routinely grown in complex SW-2 medium, containing 2% (w/v) total salts and 0.5% (w/v) yeast extract [35]. *E. coli* strains were routinely grown in complex LB medium [36]. M63 medium [37] with 20 mM glucose as the sole carbon source and NaCl ranging from 0.25 to 2.5 M, was used as minimal medium. Although *C. salexigens* can grow in M63 with 0.5 M NaCl, growth is extremely slow at this salinity (g = 24 h), and cells take a very long time to reach exponential phase. Therefore, we used M63 with 0.75 M NaCl as the standard medium for a low salt concentration in all experiments. The pH of the media was adjusted to 7.2 with KOH. Solid media contained 2% (w/v) of Bacto agar (Difco). Cultures were incubated at 37°C (*E. coli*) or 37 and 45°C (*C. salexigens*) in an orbital shaker at 200 rpm. When used, filter sterilized antibiotics were added at the following final concentrations (µg ml^−1^): ampicillin (Ap), 150 for *E. coli*; chloramphenicol (Cm), 30 for *E. coli*; gentamicin (Gm), 20 for *E. coli*, 25 for *C. salexigens*; kanamycin (Km), 50 for *E. coli* and 75 for *C. salexigens*; rifampicin (Rf), 25 for *C. salexigens*; streptomycin (Sm) 20 for *E. coli* and 50 for *C. salexigens*. Growth was monitored as the optical density of the culture at 600 nm (OD_600_) with a Perkin-Elmer Lambda 25 UV/Vis spectrophotometer.

**Table 1:** Bacterial strains and plasmids used in this study.

Strain or plasmid	Relevant genotype and/or description	Source or reference
*C. salexigens* strains		
CHR61	Spontaneous Rf^r^ mutant of *C.salexigens*DSM 3043^T^	[30]
CHR62	CHR61 Δ*ectABC*::Tn*1732*; Rf^r^ Km^r^	[30]
CHR136	CHR61 *ectD*::Ω; Rf^r^ Sm^r^ Spc^r^	[31]
CHR185	CHR61 *otsA*:: Ω; Rf^r^ Sm^r^ Spc^r^	This study
CHR186	CHR61Δ*ectABC*::Tn*1732otsA*::Ω; Rf^r^ Km^r^ Sm^r^ Spc^r^	This study
CHR187	CHR61 *ectD*::Ω *otsA*::Ω; Rf^r^ Sm^r^ Spc^r^	This study
*E. coli* strain		
DH5α	*supE44*Δ(*lac*)*U169*φ80d*lacZΔM15 hsdR17 recA1 endA1 gyrA96 thi-1 relA1*; host for DNA manipulations	[70]
Plasmids		
pKS/SK(-)	Cloning vector; Ap^r^	Stratagene
pHP45Ω	pBR322 derivative carrying the Ω cassette; Ap^r^ Sm^r^ Sp^r^	[43]
pRK600	Helper plasmid; Cm^r^ *tra*	[45]
pJQ200-SK	Suicide vector; Gm^r^ *mob sac*	[44]
pMO1	4.3-kb blunt fragment from *C. salexigens* genome (containing *otsA, Csal0235*) cloned into pKS in *Sma*I; Ap^r^	This study
pMO2	pMO1 derivative with an 1.3 fragment deleted by *Sac*I digestion; Ap^r^	This study
pMO3	pMO2 derivative with Ω casete within *otsA*; Ap^r^ Sm^r^, Spc^r^	This study
pMO4	5-kb *Apa*I-*Sac*I fragment from pMO3 (containing *otsA*, Csal0235) cloned into pJQ200-SK; Gm^r^ Sm^r^ Spc^r^	This study

### Tolerance to desiccation

Strain CHR61 was grown in M63 minimal medium with 2.5 M NaCl at 37°C or 45°C, and 1 ml aliquots of the cultures in late-exponential/early stationary phase were harvested by centrifugation. Cell pellets were washed with the same medium without any carbon source, centrifuged and, after removing the supernatant, vacuum dried. For each temperature, two variations of the protocol described by Manzanera and co-workers [38] were used. In a first step two replicates of all samples were dried by vacuum in a Memmert V0200 vacuum oven at 30°C and 313 mbar for 20 h. After that, for each sample, one replicate was taken out from the oven and stored at 28°C, and the other was subjected to a further step under vacuum consisting on a temperature ramping of 2°C/min with a 15-min pause after every increase of 2°C, up to a maximum temperature of 40°C, followed by storage at 28°C. For assessment of viability, after variable time periods 1 ml of complex medium was added to all samples, serial dilutions were plated on complex medium agar plates, incubated at 37°C and counted to determine CFU/ml. Viability was measured before (taken as 100% survival) and just after drying, and at 4 days, 1, 2, and 3 weeks storage, and expressed as percentage of viable cells. Cells of *E. coli* strain MC4100 were grown in 0.25 M NaCl M63 minimal medium at 37°C, and processed as for *C. salexigens*.

### Extraction and determination of intracellular solutes by ^13^C-NMR spectroscopy

*C. salexigens* strain CHR61 and strains CHR136 (*ectD*::Ω), CHR185 (*otsA*::Ω) and CHR187 (*ectD*::Ω *otsA*::*Ω*) were grown in M63 with 2.5 M NaCl at 45°C. *C. salexigens* strains CHR62 (Δ*ectABC*::Tn*1732*) and CHR186 (Δ*ectABC*::Tn*1732*-*otsA*::Ω) were grown in M63 with 0.75 M NaCl at 37°C. Cells cultured until late-exponential phase were collected by centrifugation and washed with the same medium without any carbon source. Extraction was performed as described by Bligh and Dyer [39]. Cell pellets from 200 ml cultures were resuspended in 10 ml of extraction mixture (methanol∶chloroform∶water; 10∶5∶4) and intracellular solutes were extracted by gently shaking for 30 min at 37°C. The cell debris was removed by centrifugation and supernatants were extracted once with chloroform∶water (1∶1) and freeze-dried. The solids were dissolved in D_2_O (0.6 ml). ^13^C-NMR spectra were recorded at 25°C on a Brucker AV500 spectrometer at 125 MHz. The chemical shifts are reported in ppm on the *δ* scale relative to tetramethylsilane. Signals were assigned by comparison with previously published chemical shift values [30], [27] or with ^13^C-NMR patterns of pure compounds.

### Extraction and determination of intracellular trehalose content

Trehalose determination was performed basically as described by Blázquez et al [40]. Cells were grown in M63 minimal medium at 37°C (0.75 M or 2.5 M NaCl) and at 45°C (2.5 M) and pellets from 15 ml of late exponential-early stationary phase cultures were washed with isotonic carbon-free medium and resuspended in 1 ml of the same medium. Cells were lysed by 30 min incubation at 95°C and, after centrifugation, the supernatant was used to determine the trehalose content in a total volume reaction of 200 µl. This reaction contained 100 µl of the supernatant, 90 µl of 25 mM sodium acetate buffer (pH 5.6) and 0.02 U of commercial trehalase (Sigma). For each culture sample, endogenous glucose content was monitored by performing a parallel reaction in which the trehalase solution was substituted by water. After overnight incubation at 37°C, the glucose released by trehalose hydrolysis was determined by adding 150 µl of the previous reaction to 150 µl of mixture 0.66 mg ml^−1^ *Aspergillus niger* glucose oxidase (Sigma) and 0.25 mg ml^−1^ horseradish peroxidase in 0.5 M phosphate buffer, pH 6.0 (Sigma) and 50 µl of 2.33 mg ml^−1^ o-toluidine (Panreac). After 30 min of incubation at 37°C, 1.5 ml of water was added to the samples and absorption was measured at 420 nm in a Perkin Elmer Lambda 25 UV/Vis spectrophotometer. The values were compared to those obtained from stock solutions of glucose standards within a concentration range of 0 to 300 µg ml^−1^. Finally, trehalose content was calculated from the glucose content by performing a standard curve with commercial trehalose (Sigma) at concentrations ranging from 1 to 5 mM. Trehalose concentration was expressed as µmol mg protein^−1^.

### LC-MS quantification of ectoine and hydroxyectoine

1 ml of cells grown until late exponential phase were collected for liquid chromatography-mass spectrometry (LC-MS) analysis of intracellular content of ectoine and hydroxyectoine. The solutes were extracted by using a modified Bligh and Dyer technique [39] as described by Kraegeloh and Kunte [41]. Chromatographic separation and MS quantification of ectoine and hydroxyectoine was performed as described by Argandoña et al. [42]. Solute concentration was expressed as µmol/mg protein.

### Determination of protein content

Cell protein content was determined by triplicate by using BCA Protein Assay kit (PIERCE) as described by García-Estepa et al. [31].

### Methods for nucleic acid manipulation and constructions of mutant strains

Plasmid DNA was isolated from *E. coli* with a Wizard Plus SV miniprep kit (Promega) and genomic DNA was isolated with a SpinClean Genomic DNA Purification kit (Mbiotech). Restriction enzyme digestion and ligation were performed as recommended by the manufacturers (Amersham-Pharmacia Biotech and Fermentas). DNA sequencing was performed by Newbiotechnic (Seville, Spain).

**Figure 1:**
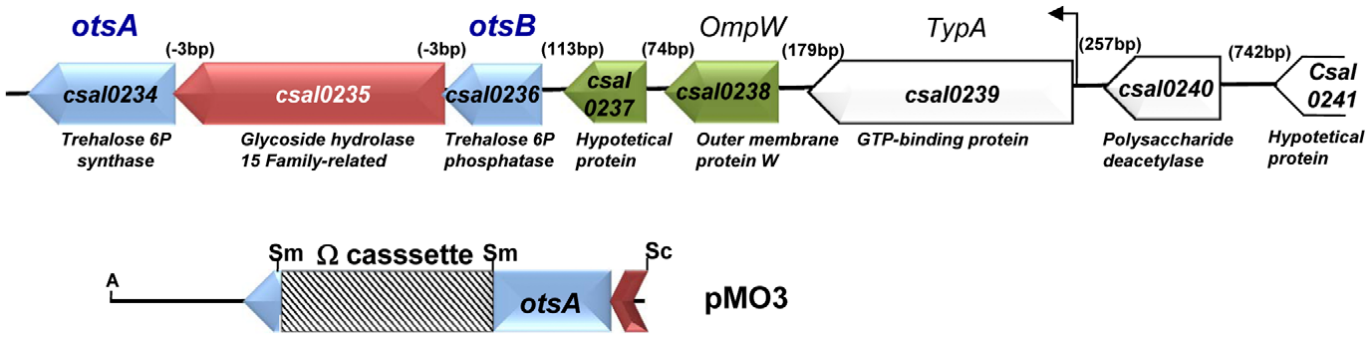
OtsA is involved in trehalose synthesis by *C. salexigens*. Genetic *con*text of the *C. salexigens treha*lose synthesis genes *otsA* (encoding a trehalose-6-phosphate synthase) and *otsB* (encoding a trehalose-6-phosphate phosphatase). Numbers into brackets denote intergenic regions. The arrow upstream of *csal0239* denotes a predicted α^70^-dependent promoter. For the generation of *otsA*::Ω mutant strains, *otsA* was inactivated by the insertion of an Ω cassette, which carries resistance genes for streptomycin/spectinomycin, into its unique site *Sma*I (Sm), giving the plasmid pMO3.

To construct *C. salexigens otsA* mutants, a 4,257-bp fragment from the *C. salexigens* genome containing the *otsA* gene and 1,683 bp from the adjacent gene *csal0235* was amplified with *Pfu* Turbo DNA polymerase (Stratagene) by using the primers *otsA* ^C^-FW (5′-AACCGAGTTCTTCGTACACCTGTTCCTTG-3′) and *otsA* ^C^-RV (5′-TTCGACGGCGATCCCTTCTTCTCG-3′). The amplified PCR fragment was cloned into pBluescript KS digested with *Sma*I to obtain the plasmid pMO1. This plasmid was digested with *Sac*I and religated to delete a 1.3-kb fragment, resulting in the plasmid pMO2. Subsequently, a *Sma*I recognition site was generated in *otsA* gene, using the PCR-based QuickChange Site Directed Mutagenesis Kit (Stratagene) and the primers *otsA* ^c^-*Sma*I-FW (5′-CGAACTCCTTGCAG**ACCCGGG**TCATGCCGTCGCGC-3′) and *otsA* ^c^-*Sma*I-RV (5′-GCGCGACGGCATGA**CCCGGG**TCTGCAAGGAGTTCG-3), that were modified (residues underlined) to result in the corresponding restriction site (in bold). The obtained plasmid was linearized with *Sma*I and ligated to a 2-kb *Sma*I fragment from pHP45-Ω [43], containing the Ω interposon used for insertional mutagenesis (Sm^r^) in order to generate the plasmid pMO3 (see Fig. 1). A 5-kb *Sac*I-*Apa*I fragment containing the Ω cassette insertion in *otsA* gene from pMO3 was cloned into the suicide vector pJQ200-SK (Gm^r^) [44] and the plasmid generated (pMO4) was transferred to *C. salexigens* CHR61 (wild type), CHR62 (Δ*ectABC*::Tn*1732*) and CHR136 (*ectD*::Ω) by triparental mating. Recombinant strains resulting from a double homologous recombination event were identified as Sm^r^ Gm^s^ colonies on SW-2 plates containing 10% sucrose. Insertion of the Ω cassette in these strains was confirmed by PCR and sequencing. Three of these colonies were selected for further analysis and named CHR185 (*otsA*::Ω), CHR186 (Δ*ectABC*::Tn*1732 otsA*::Ω) and CHR187 (*ectD*::Ω *otsA*::Ω), respectively. The lack of trehalose in all *otsA* mutants was confirmed by ^13^C-NMR.

### Conjugal transfer of plasmids

Plasmids were transferred from *E. coli* to *C. salexigens* by triparental mating on SW-2 medium, using pRK600 [45] as a helper plasmid, as described by Vargas et al. [46].

### DNA and protein sequences analysis

Sequence of the *C. salexigens* DSM 3043 genome is available at JGI http://genome.jgi.doe.gov/pages/home.jsf?query=chromohalobacter&searchType=keyword. Sequence data were analyzed using BLAST (http://ncbi.nlm.nih.gov/BLAST). Conserved domains within proteins were identified at NCBI's Conserved Domain Database (http://www.ncbi.nlm.nih.gov/Structure/cdd/cdd.shtml) [47]. Promoter sequences were predicted using BGDP Neural Network Promoter Prediction (http://www.fruitfly.org/seq_tools/promoter.html).

### RNA extraction, Reverse Transcriptase-PCR, and Real Time PCR

*C. salexigens* cultures were grown in M63 at 37°C (with 0.75 M or 2.5 M NaCl) and at 45°C (2.5 M NaCl) until late exponential phase and cells were harvested by centrifugation. Total RNA was extracted with the High Pure RNA isolation kit (Roche) according to the manufacturer's instructions, which includes a step to remove directly in the column the chromosomal DNA by DNase digestion. After isolation, the integrity of the RNA samples was assessed by agarose gel electrophoresis and the absence of DNA contamination was checked by PCR using the 16SrRNA primers *16S-RT-fw* and *16S-RT-rv* [42]. The RNA concentration was determined spectrophotometrically at 260 nm and RNA was stored at −80°C until use. cDNA was synthesized by using the Transcriptor First Strand cDNA synthesis kit (Roche) following the manufacturer's recommendations. 2 µg of total RNA was previously denatured at 65°C for 10 min, and then random hexamers (60 µM), protector RNAse inhibitor (20 U), reverse transcriptase (10 U) and reaction buffer were added to a final volume of 20 µl. The reaction was incubated at 25°C for 10 min followed by 60 min-incubation at 50°C. Finally, the mixture was incubated 5 min at 85°C to inactivate the RT enzyme. Synthesized cDNA was kept at −20°C until use.

To check if *csal0234*(*otsA*)-*csal0235*-*csal0236*(*otsB*)-*csal0237*-*csal0238*-*csal0239*-*csal0240* are co-transcribed, intergenic regions were amplified by using as a template 2 µl of *C. salexigens* cDNA synthesized from RNA isolated from cells grown in M63 with 2.5 M NaCl at 37°C. The cycling conditions for the PCR reactions were as follows: 2 min at 98°C, followed by 35 cycles of 30 sec at 98°C, 30 sec at 60°C, and 30 sec to 1 min (depending on the amplicon size) at 72°C, followed by 10 min extension at 72°C, and the primer pairs included in Table S1.

For real-time PCR, one set of specific primers that amplified an internal region of *otsA* were used and designed with the Primer3 software [48]. These were *otsA-qRT-fw* (5′-TCTTCCTGCACATTCCCTTTCC-3′) and *otsA-qRT-rv* (5′-GCGATCGTTTTCGGTCTGAAAG-3′) which have a length of 22 pb, a G/C content over 50% and a Tm of 60.3°C and amplified a PCR product of 120 pb length. Secondary structures and dimer formation were controlled using Sygma-Aldrich web analyzer. Real-time PCR was performed as described by Argandoña et al. [42] in 96-well plates using an ABI Prism 7000 sequence detector (Applied Biosystems) and the FastStart Master (Rox, Roche). Amplification data were analyzed with the ABI Prism 7000 software (Applied Biosystems). Transcripts levels were calculated by the 2^−ΔCt^ method using the mRNA levels of *16S rRNA* gene as an endogenous control (housekeeper) to normalize the data obtained within each sample. As a control condition, we selected 0.75 M 37°C (low salt and optimal temperature). In consequence, the mRNA levels of *otsA* gene in all conditions tested was referenced to the levels observed in 0.75 M 37°C.

### Nucleotide sequence accession numbers

The *C. salexigens* genome sequence is available at Gene bank under the accession NC_007963. Accession numbers for the genes *csal0234* (*otsA*), *csal0235*, and *csal0236* (*otsB*) are YP_572297, YP_572298, and YP_572299, respectively.

## Results

### Inactivation of *C. salexigens otsA* gene suppresses trehalose synthesis from glucose

Previous studies by our group revealed that trehalose is accumulated in *C. salexigens* in response to heat stress or when ectoines are absent [30], [31].To gain insight into the role of trehalose in the response to abiotic constrains in this microorganism, we first inspected the *C. salexigens* genome searching for genes involved in trehalose synthesis. Out of the five different biosynthetic pathways for trehalose described so far, only the pathway encoded by the *otsAB* genes was found in *C. salexigens*. The corresponding genes (*otsA, csal0234*, and *otsB, csal0236*) laid at the 3′-end of a set of eight genes (*csal0242* to *csal0234*) all oriented in the same direction in the complementary strand (Fig. 1). *otsA* and *otsB* and were separated by *csal0235*, encoding a putative hydrolase of the family 15 of glycosyl hydrolases that was phylogenetically close to characterized trehalases and trehalose-6P-hydrolases from related bacteria (data not shown). There are not intergenic regions between *otsB* and *csal0235*, or between *csal0235* and *otsA*, suggesting that they form one operon. This was confirmed by amplification of the *otsA-csal0235*, *csal0235*-*otsB*, and *otsB*-*csal0237* intergenic regions by using RT-PCR (Fig. S1B). In contrast, amplification of the *csal0237*-*csal0238*, and *csal0239*-csal0240 intergenic region gave negative results (Fig. S1B). Amplification of the *csal0238*-*csal0239* resulted in a very tiny band (Fig. S1B, lane 2). However, expression of *csal0238*, encoding a putative outer-membrane protein W (OmpW), was very low under the conditions used (Fig. S1D), opening the possibility that *csal0238 and csal0239* might be co-transcribed. Computer-assisted analysis did not reveal any clear promoter upstream of *csal0237* or *csal0238*, but predicted a putative σ^70^-dependent promoter upstream of *csal0239*.

**Figure 2:**
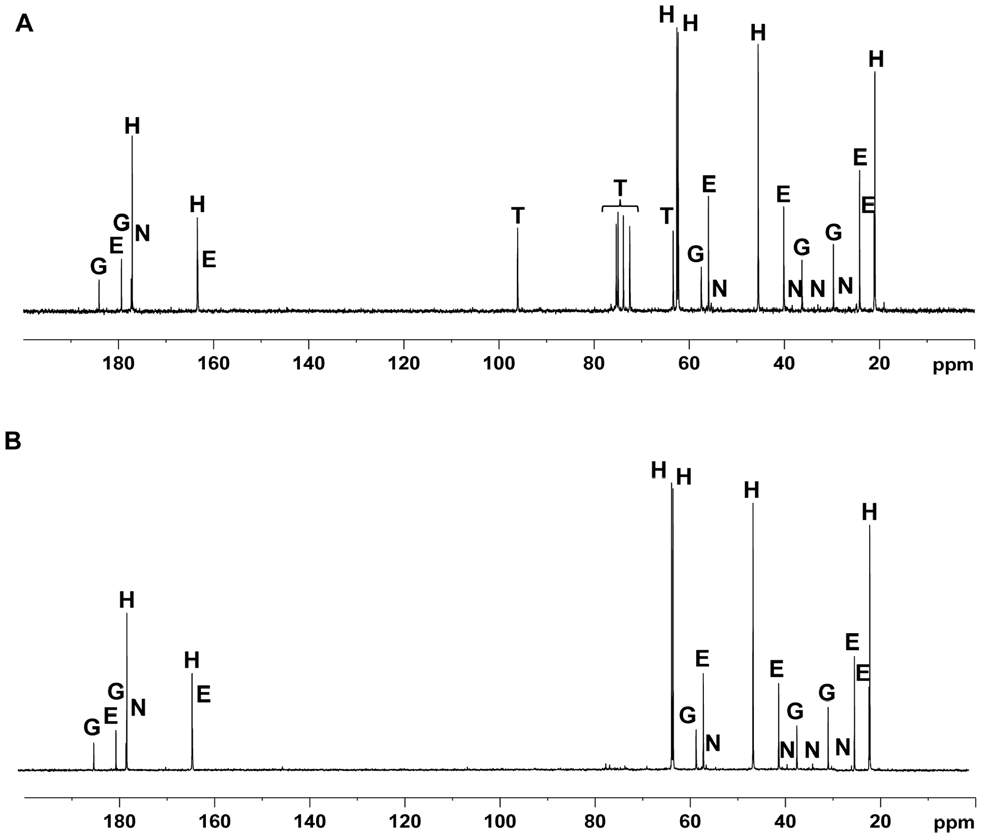
Natural abundance ^13^C-NMR spectrum of major cytosolic solutes of the *C. salexigens* wild-type and *otsA* strains. CHR61control (A) and *otsA*:: (CHR185) (B) strains were grown in M63 minimal medium at 45°C with 2.5 M NaCl. Cells were extracted as described in Materials and Methods. The major solutes were hydroxyectoine (H), ectoine (E), trehalose (T), glutamate (G) and Nγ-acetyldiaminobutyric acid (N).

*C. salexigens* OtsA and OtsB showed about 50% of identity to orthologous proteins from *Burkholderia*, *Ralstonia*, *Halorhodospira halophila* or *Pseudomonas stutzeri*. All residues corresponding to the active site of OtsA proteins were conserved in *C. salexigens* OtsA, as determined by the alignment of this protein with the well studied *E. coli* trehalose-6-P synthase [49], [50] (data not shown). To confirm that in *C. salexigens* the *otsAB*-encoded pathway is involved in trehalose synthesis from glucose, we constructed an *otsA*::Ω mutant (strain CHR185) and measured its ability to synthesize trehalose in minimal medium M63, compared to the CHR61 strain. Figure 2 shows the natural abundance ^13^C-NMR spectra of the CHR61 and *otsA*::Ω strains grown at 45°C in glucose minimal medium with 2.5 M NaCl. The spectrum of the *C. salexigens* CHR61 strain contained five sets of resonances that were assigned to hydroxyectoine, ectoine, trehalose, glutamate and the ectoine precursor N-γ-acetyldiaminobutyric acid (Fig. 2A). However, chemical shifts corresponding to trehalose were absent from the spectrum of the *C. salexigens otsA*::Ω strain (Fig. 2B). These data demonstrate that OtsA is indeed responsible for the synthesis of trehalose from glucose in *C. salexigens*.

### Transcriptional and post-transcriptional regulation of trehalose synthesis by salinity and temperature stress

**Figure 3:**
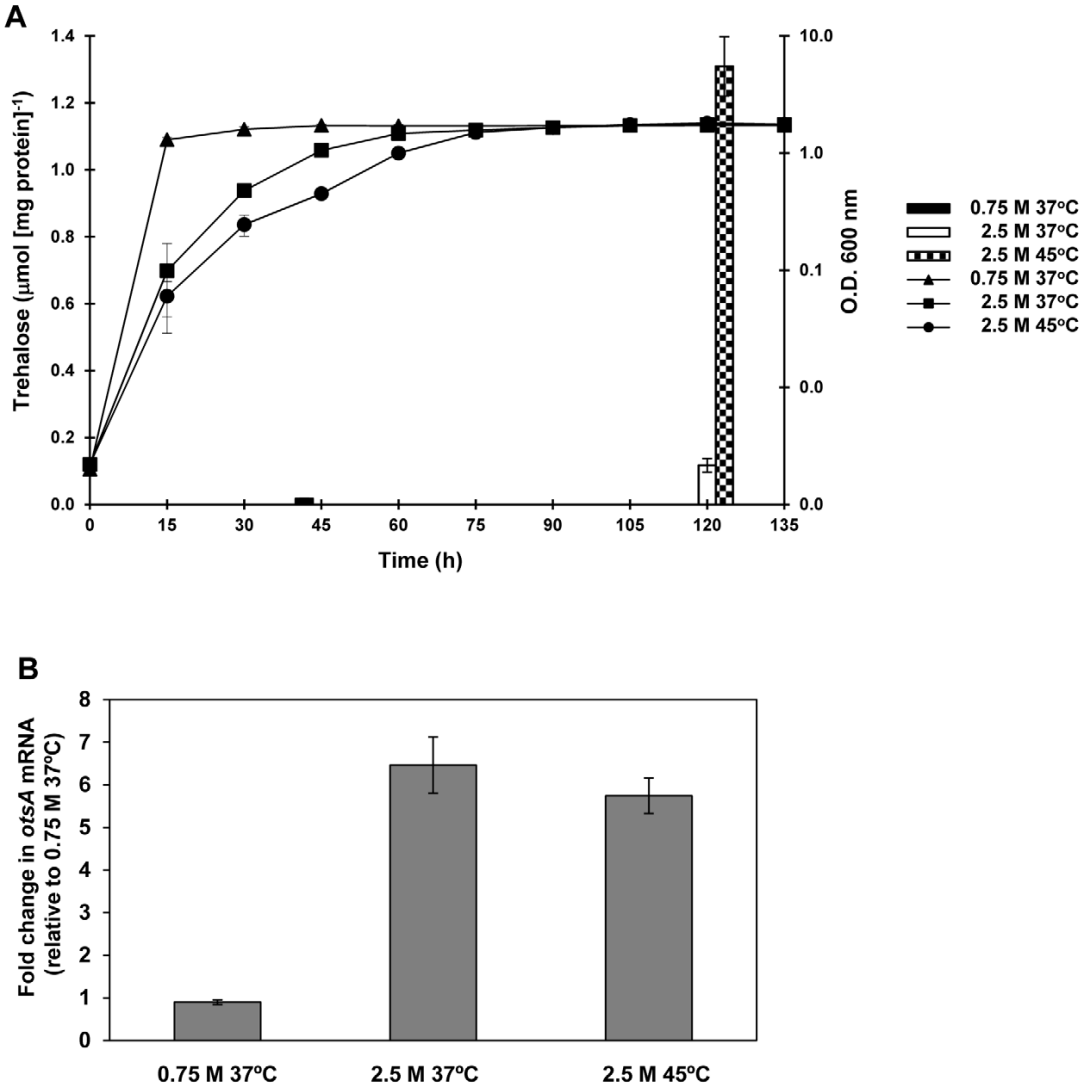
Regulation of trehalose synthesis by *C. salexigens* in response to temperature and salinity stress. (A) Accumulation of trehalose by *C. salexigens* CHR61 in response to temperature and salinity. Cells were grown in minimal medium M63 at 37°C (with 0.75 and 2.5 M NaCl) and 45°C (with 2.5 M NaCl), up to early stationary phase. Trehalose content was measured colorimetrically as described in Materials and Methods. For each determination, a growth curve under the same condition used to measure trehalose accumulation is shown. Histograms representing trehalose accumulation are shown above the sampling time. The trehalose values are the mean of three replicas of each condition in two independent experiments ± SD (standard deviation). *C. salexigens* does not grow at 45°C with 0.75 M NaCl. (B) relative *otsA* mRNA levels determined by quantitative PCR (qPCR) in *C. salexigens* CHR61 strain. Total RNA was extracted from cells grown in minimal medium M63 under the same conditions of temperature and salinity as above, and *otsA* mRNA abundance was measured by quantitative PCR as described in Materials and Methods. The data are expressed in relative units and were estimated by the 2^−ΔCt^ method using the 16S rRNA gene as an internal control to normalize expression in each sample. Results are shown as the fold change in expression relative to that of 0.75 M 37°C. Real-time PCR quantification was performed twice, using RNA samples from independent cultures, and the values are the means standard deviations of three replicates from two independent experiments.

By using ^13^C-MNR, we previously observed that trehalose accumulation in *C. salexigens* is switched-on when cells are grown at 45°C [31]. In this work, we have quantified trehalose accumulation under different conditions of salinity and temperature, by using a colorimetric approach (see Materials and Methods). To determine trehalose content in response to salinity, we compared trehalose accumulation at 0.75 or 2.5 M NaCl in cells grown at optimal temperature (37°C). At this temperature, trehalose concentration increased about 2.8-fold in response to salinity stress, but the total amount accumulated (ca. 0.05 µmol/mg protein) remained too low as to counterbalance the external salinity (Fig. 3A). *C. salexigens* cannot grow at 45°C with 0.75 M NaCl [31]. Thus, to determine trehalose content in response to temperature stress, we compared the accumulation of trehalose at 37°C and 45°C in cells grown at 2.5 M NaCl. As shown in Figure 3A, trehalose accumulation by *C. salexigens* cells grown at this salinity increased by 12-fold from 37°C to 45°C. These data confirm our previous finding that in *C. salexigens* trehalose biosynthesis is mainly triggered by heat stress.

To determine if this temperature-dependent trehalose synthesis was transcriptionally controlled, the *otsA* mRNA abundance was measured by qPCR at the same conditions as above. As illustrated in Figure 3B, a ca. 6.5-fold increase of *otsA* relative levels of transcripts was found from 0.75 M to 2.5 M NaCl. However, no further temperature-dependent induction of the *otsA* mRNA levels was observed when cells were grown at 45°C and 2.5 M NaCl. These results, taken together, suggest that trehalose synthesis is transcriptionally regulated by osmotic stress, and post-transcriptionally regulated by heat stress.

### Role of trehalose as a secondary osmolyte involved in osmo- and thermoprotection of *C. salexigens*

**Figure 4:**
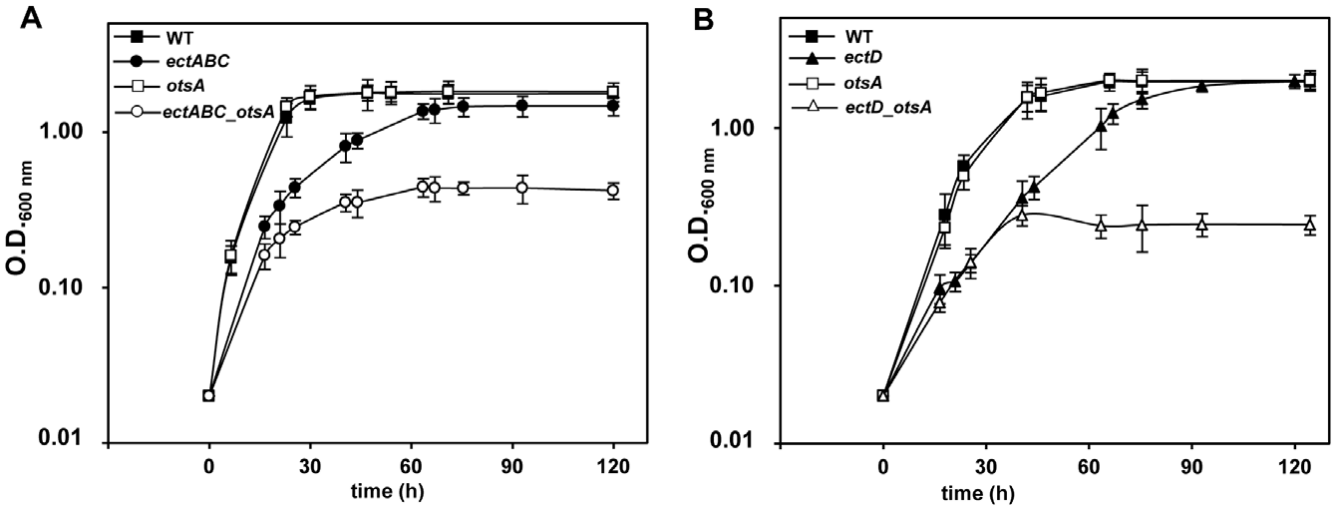
Contribution of trehalose to the salinity and high temperature tolerance of *C. salexigens*. (A) Effect of salinity on the growth of *C. salexigens* ectoines and/or trehalose defective mutants. Cells of *C. salexigens* CHR61 (▪), Δ*ectABC*::Tn*1732* (•), *otsA*::Ω (□) and Δ*ectABC*::Tn*1732 otsA*::Ω (○) strains were grown at 37°C in M63 minimal medium with 0.75 M NaCl. (B) Effect of temperature on the growth of *C. salexigens* hydroxyectoine and/or trehalose defective mutants. Cells of *C. salexigens* CHR61 (▪), *ectD*::Ω (▴), *otsA*::Ω (□), and *ectD*::Ω *otsA*::Ω (▵) strains were grown at 45°C in M63 minimal medium with 2.5 M NaCl. Values shown are the mean of two replicas of each condition in three independent experiments ± SD (standard deviation).

In *C. salexigens*, the main compatible solutes involved in osmo- and thermo-protection are ectoine and hydroxyectoine, respectively [29]. No differences were observed in the growth of CHR61 and the *otsA*::Ω mutant at 37°C with 0.75 to 2.5 M NaCl (data not shown). To test if trehalose confers osmoprotection in the absence of ectoines, a double ectoine(s)- and trehalose-deficient strain (Δ*ectABC*::Tn*1732 otsA*::Ω) was constructed, and its growth was compared to that of the wild type and *otsA*::Ω mutant at 37°C with 0.75 M NaCl. Despite the concentration of trehalose is very low at this salinity (see Fig. 3A), these conditions were chosen as the ectoine(s)-deficient strain CHR62 cannot grow at 37°C above 0.75 M NaCl (nor at 45°C at any salinity) [30], [31]. As shown in Figure 4A, the growth of the *C. salexigens otsA*::Ω single mutant was indistinguishable from that of the CHR61 strain and the Δ*ectABC*::Tn*1732* mutant displayed a delayed growth. In contrast, growth of the double Δ*ectABC*::Tn*1732 otsA*::Ω strain was severely impaired under these conditions, as its culture could not reach an OD_600_ above 0.45 units.

To check the contribution of trehalose to the thermotolerance of *C. salexigens*, a double mutant showing a reduced hydroxyectoine synthesis (*ectD*::Ω) and a null trehalose synthesis (*otsA*::Ω) was constructed, and its growth at 45°C with 2.5 M NaCl compared to that of CHR61 and single *ectD*::Ω and *otsA*::Ω strains. These are conditions where the accumulation of both hydroxyectoine [31] and trehalose are maximal in the control strain (see Fig. 3). Figure 4B shows that growth of the *otsA*::Ω mutant: was not affected under these conditions, and growth of the *ectD*::Ω mutant was delayed, whereas growth of the double mutant *ectD*::Ω *otsA*::Ω was severely impaired. These data suggest that trehalose is a secondary compatible solute involved in. *salexigens* tolerance to salinity and high temperature.

### Desiccation-tolerance of *C. salexigens*

Since both hydroxyectoine and trehalose contribute to thermoprotection of control strain CHR61, we investigated whether these compatible solutes are also involved in the tolerance of *C. salexigens* to desiccation. In a first step, the intrinsic tolerance of *C salexigens* CHR61 to desiccation was measured in cells grown in M63 with 2.5 M NaCl, at 37°C or 45°C, and compared to that of *E. coli* MC4100 grown at 37°C in the same medium with 0.25 M NaCl (Table 2). For this purpose, we used two variants of the protocol described by Manzanera and co-workers for *E. coli* [38], vacuum-drying and vacuum-drying with increasing temperature. For *E. coli* MC4100, survival after vacuum-drying was of 58%, and decreased to about 47% after 4 days of storage at 28°C, followed by gradually lower survival levels to ca. 12% after 3 weeks. Compared to the drying treatment, the drying+high temperature protocol did not influence cell survival of *E. coli*. Remarkably, *C. salexigens* CHR61 showed very low survival levels after vacuum-drying, although viability of cells grown at 45°C was 2-fold higher than viability of cells grown at 37°C (ca. 4.6 and 1.8%, respectively). Interestingly, for cells grown at 37°C (but not at 45°C), application of a gradual increase of temperature during drying increased cell survival by 4.5-fold (ca. 9%). To check if this was related to trehalose and/or hydroxyectoine synthesis, we measured trehalose and ectoines (ectoine and hydroxyectoine) levels in wild type cells grown at 37°C before and after the two drying treatments. However, we did not find significant differences (data not shown). Regardless of the drying process, *C. salexigens* survival rates were extremely low after 4 days storage. Under conditions of maximal hydroxyectoine and trehalose synthesis (45°C), the *ectD*::Ω and *otsA*::Ω single mutants showed very low survival rates after vacuum-drying (<0.5%), suggesting that trehalose and hydroxyectoine might be responsible for the desiccation tolerance levels showed by the wild type grown at high temperature.

**Table 2:** Survival of *E. coli* and *C. salexigens* strains after vacuum-drying and subsequent storage at 28°C.

	*E. coli* (37°C)		Wild type (37°C)		Wild type (45°C)		*ectD*::Ω (45°C)	*otsA*::Ω (45°C)
Sample	V^1^	V+T^2^	V	V+T	V	V+T	V	V
Initial	100	100	100	100	100	100	100	100
After drying	58±3.38	27.98±9.40	1.78±0.40	9.02±1.33	4.63±0.2	0.6±0.4	0.451±0.05	0.093±0.02
After storage for:								
4 days	47.2±0.48	23.13±12.7	0.16±0	0.069±0	0.067±0.81	0.026±0	0.034±0	0.046±0
1 week	40.44±7.26	21.12±4.81	0.0018±0	0	0.0067±0	0.011±0	0	0
2 weeks	23.33±0.67	16.12±8.61	0	0	<0.001	0	0	0
3 weeks	12.45±2.59	9.97±5.09	0	0	0	0	0	0

The values are means ± standard errors.

1 Desiccation under vacuum.

2 Desiccation under vacuum with temperature ramping.

## Discussion

*C. salexigens* finely adjusts its cytoplasmic compatible solute pool in order to cope with high salinity and supra-optimal temperatures [29]. This is achieved by a highly hierarchical accumulation of solutes, dominated by the uptake of external osmoprotectants such as glycine betaine or its precursor choline [27], [33], and followed by the synthesis of endogenous solutes, mainly ectoines (ectoine and hydroxyectoine), and minor amounts of glutamate, glutamine, trehalose and glucosylglycerate. Among the endogenous compatible solutes, ectoine and hydroxyectoine are the main responsible for osmo- and thermo- tolerance, respectively [29], [31].

Most studies about compatible solute synthesis and its regulation focused on ectoines and glycine betaine [10], [29] but very little is known about the role of trehalose in *C. salexigens* stress response. We previously showed that in *C. salexigens* trehalose synthesis is triggered up by high temperature [31] or when ectoine synthesis is impaired [30]. In this work, we demonstrate that *C. salexigens* uses trehalose as a secondary compatible solute involved in osmo- (at least at low salinity) and thermoprotection. Very interestingly, *C. salexigens* is able to synthesize another sugar-type compatible solute, glucosylglycerate, especially under low salinity conditions [33] or in an ectoine-deficient mutant [34]. Glucosylglycerate is also accumulated in the extremely halophilic methanogenic archaeon *Metanohalophilus portucalensis* and the cyanobacterium *Synechococcus* sp. PCC 7002 growing under nitrogen-limiting conditions [8], and was shown *in vitro* to protect a number of enzymes against thermal denaturation [51]. However, glucosylglycerate was not detected neither in extracts of the wild type grown at high salinity [33] or a combination of high salinity and high temperature (see Fig. 2A), nor in extracts of the *otsA*::Ω (Fig. 2B) or the *ectD*::Ω (not shown) mutants grown under high salinity and high temperature conditions. These extracts contained similar amounts of glutamate as the wild type strain. In summary, we could not find evidence for other solutes (i.e. glutamate, glucosylglycerate) substituting or compensating the lack of trehalose or the reduction of hydroxyectoine levels in the *otsA*::Ω and *ectD*::Ω mutants.

The role of trehalose as an osmo- and thermoprotectant has been suggested in several mesophilic organisms such as yeasts [11], [52], *E. coli* [15] and *Salmonella enterica* serovar Typhimurium [53]. In contrast, in *C. salexigens* (this work) and most other halophilic heterotrophic aerobic bacteria analyzed [54], trehalose is always found as a minor product compared to ectoine and/or hydroxyectoine. The fact that trehalose concentration in *C. salexigens* never exceeds 1.5 µmol/mg protein, whereas ectoine or hydroxyectoine can reach much higher levels [31], fits well with this accessory role for trehalose. Interestingly, thermophilic bacteria such as *T. thermophilus* [55] and hyperthermophilic archaea as *Pyrococcus horikoshii* and *Thermococcus* [5] accumulate trehalose in response to osmotic stress. In these species, however, trehalose does not seem to be involved in thermotolerance. Instead, specific compatible solutes, such as mannosylglycerate and di-*myo*-inositol-phosphate, were proposed to be involved in thermoprotection [5], [8].

Our data suggest that *csal0237*-*otsB*-*csal0235*-*otsA* belong to the same operon, and therefore have the same regulatory pattern. Very interesting, *csal0235* is phylogenetically close to characterized trehalases and trehalose-6P-hydrolases from related microorganisms (not shown), suggesting a role of *Csal0235* in trehalose metabolism in *C. salexigens*. This will be investigated in a further work. In *E. coli* and *S. enterica* serovar Typhimurium, trehalose is accumulated in response to osmotic and heat stresses, and transcription of the *otsAB* genes for trehalose synthesis is both osmo- and thermoregulated [15], [53], [56]. In contrast, despite the fact that *otsA* expression is osmoregulated, but not thermoregulated (see Fig. 3B), trehalose is accumulated by *C. salexigens* mainly in response to heat stress. These findings, which suggest that trehalose synthesis in *C. salexigens* is primarily regulated by temperature at the post-transcriptional level, fit well with a role of trehalose as an secondary heat stress protectant in *C. salexigens*. Thus, besides conferring osmoprotection, transcriptional regulation of trehalose synthesis by salinity might be viewed as a long-term mechanism necessary to achieve enough trehalose synthesis enzymes, to be quickly activated under heat stress.

Bacteria exhibit a variable degree of desiccation tolerance, and only relatively few genera are recognized as able to survive total desiccation (i.e., become anhydrobiotic), the major exceptions being Cyanobacteria [57] and spores. Our data suggest that *E. coli* is more tolerant to drying and storage than *C. salexigens*. These differences might reflect the different habitats in which these microorganisms live and thrive. Thus, the primary habitat of *E. coli* is the vertebrate gut, but it can persist for varying periods of time, in soil, manure and water [58]. In contrast, *C. salexigens* was isolated from saline water in a saltern, one habitat constrained mainly by saline and heat stresses [59].

The accumulation of trehalose in *E. coli* has been first demonstrated by the pioneering work of Larsen et al. [60] and Welsh et al. [61]. Thus, under similar conditions to those used in this work (batch cultures in M63), *E. coli* accumulated ca. 0.25 and 0.49 µmol trehalose/mg protein at 0.2 and 0.4 M NaCl, respectively [60]. On the other hand, by using batch cultures in a 25 l fermenter in modified Evan's medium with glucose, Welsh et al. reported maximal levels of ca 1.5 µmol trehalose/mg protein at 0.5 M NaCl [61]. In this work, we found that *C. salexigens* grown at high salinity accumulates ca. 0.15 and 1.3 µmol trehalose/mg protein at 37 and 45°C, respectively. Thus, the fact that *E. coli* is more desiccation-tolerant than *C. salexigens* cannot be explained by a higher trehalose content in the first one. Likewise, *Pseudomonas putida* cells that were genetically engineered to accumulate high intracellular trehalose concentrations did not show improved desiccation tolerance [62]. Therefore, at least for some populations of microorganisms, trehalose does not appear to provide full protection against desiccation damage, even when present at high concentrations. As suggested by Potts [57], desiccation is one of the most harmful stresses for microorganisms, and desiccation-tolerance involves complex structural, physiological and molecular mechanisms not yet completely understood. Whereas much has been (rightly) attributed to the protective effect of compatible solutes such as trehalose or hydroxyectoine [63]–[67], other important and alternative mechanisms that contribute to the tolerance to desiccation stress need to be elucidated. For instance, recent work on the transcriptional response of soil bacteria such as *Bradyrhizobium japonicum* [68], or the actinomycete *Rhodococcus jostii* RHA1 [69] to desiccation stress revealed, apart from compatible solutes and polysaccharide synthesis, the importance of specific transcriptional regulators, as well as systems involved in protection of cell membrane, DNA recombination and repair, stability and integrity of proteins, and oxidative stress response.

The findings that (i) *C. salexigens* cells that were pre-conditioned by heat stress to accumulate high levels of hydroxyectoine and trehalose showed an increased survival to drying, and (ii) single trehalose-deficient or hydroxyectoine-reduced mutants grown at 45°C were sensitive to drying, suggest a role of trehalose and hydroxyectoine in the tolerance of *C. salexigens* to desiccation. However, given the low survival rates of the wild type after drying, the role the above compatible solutes in the response of *C. salexigens* to desiccation stress cannot be definitively concluded.

One of the most striking findings of this work was that application of a gradual temperature ramping during drying of cells grown at 37°C improved *C. salexigens* desiccation tolerance. However, this was not correlated with a higher hydroxyectoine and/or trehalose content after drying, suggesting that this temperature-induced mechanism of desiccation tolerance is independent of the synthesis of these compatible solutes. It is possible that under the conditions tested drying was so rapid as to preclude any metabolic response (i.e. compatible solute synthesis). However, less drastic drying experiments (i.e.. slow air-drying) performed in our laboratory did not led to increased desiccation tolerance of *C. salexigens* (not shown). One possible explanation for these findings is temperature involvement in survival during desiccation through the phase change of membranes during drying and rewetting, leading to the loss of membrane integrity. The logical interpretation of this process would be that a progressive increase in drying temperature prevents membrane transition, positively affecting survival during desiccation. If so, physiological responses such as the production of heat-shock proteins and chaperones that stabilize the membrane could contribute to membrane integrity during desiccation.

This work represents an important contribution to our understanding of stress response in *C. salexigens* with respect to trehalose synthesis. The next challenge will be to characterize the *C. salexigens* genes for trehalose transport and degradation, and to test if exogenously added trehalose and/or hydroxyectoine improve desiccation tolerance in this extremophilic microorganism.

## Supporting Information

Figure S1 **Genetic organization of** ***C. salexigens*** **trehalose synthesis genes** ***otsA*** **and** ***otsB*** **.** Amplification of intergenic regions between *csal240* and *csal239* (lane 1), *csal239* and *csal238* (lane 2), *csal238* and *csal237* (lane3), *csal237* and *otsB* (lane 4), *otsB* and *csal235* (lane 5) and *csal235* and *otsA* (lane 6) genes by PCR using *C. salexigens* genomic DNA as template (A) or by RT-PCR (B). Intragenic regions of *csal240*, *csal239*, *csal238*, *csal237*, *otsB*, *csal235* and *otsA* genes were amplified by PCR using *C. salexigens* genomic DNA as template (C) or by RT-PCR (D), as a positive control of individual gene expression. cDNA was synthesized from RNA isolated form cultures of *C. salexigens* grown in M63 at 37°C with 2.5 M NaCl. M, molecular weight marker (1 kb ladder, Invitrogen).
(PDF)

Table S1 **List of primers for RT-PCR assay.**
(PDF)
