# Supplementary material for: Role of Trehalose in Salinity and Temperature Tolerance in the Model Halophilic Bacterium Chromohalobacter salexigens
Source: PLoS One. 2012 Mar 20;7(3):e33587. doi: 10.1371/journal.pone.0033587 (PMC3308980; doi:10.1371/journal.pone.0033587)
Supplement: Figure S1 — Genetic organization of C. salexigens trehalose synthesis genes otsA and otsB . Amplification of intergenic regions between csal240 and csal239 (lane 1), csal239 and csal238 (lane 2), csal238 and csal237 (lane3), csal237 and otsB (lane 4), otsB and csal235 (lane 5) and csal235 and otsA (lane 6) genes by PCR using C. salexigens genomic DNA as template (A) or by RT-PCR (B). Intragenic regions of csal240, csal239, csal238, csal237, otsB, csal235 and otsA genes were amplified by PCR using C. salexigens genomic DNA as template (C) or by RT-PCR (D), as a positive control of individual gene expression. cDNA was synthesized from RNA isolated form cultures of C. salexigens grown in M63 at 37°C with 2.5 M NaCl. M, molecular weight marker (1 kb ladder, Invitrogen). (PDF) [file pone.0033587.s001.pdf]

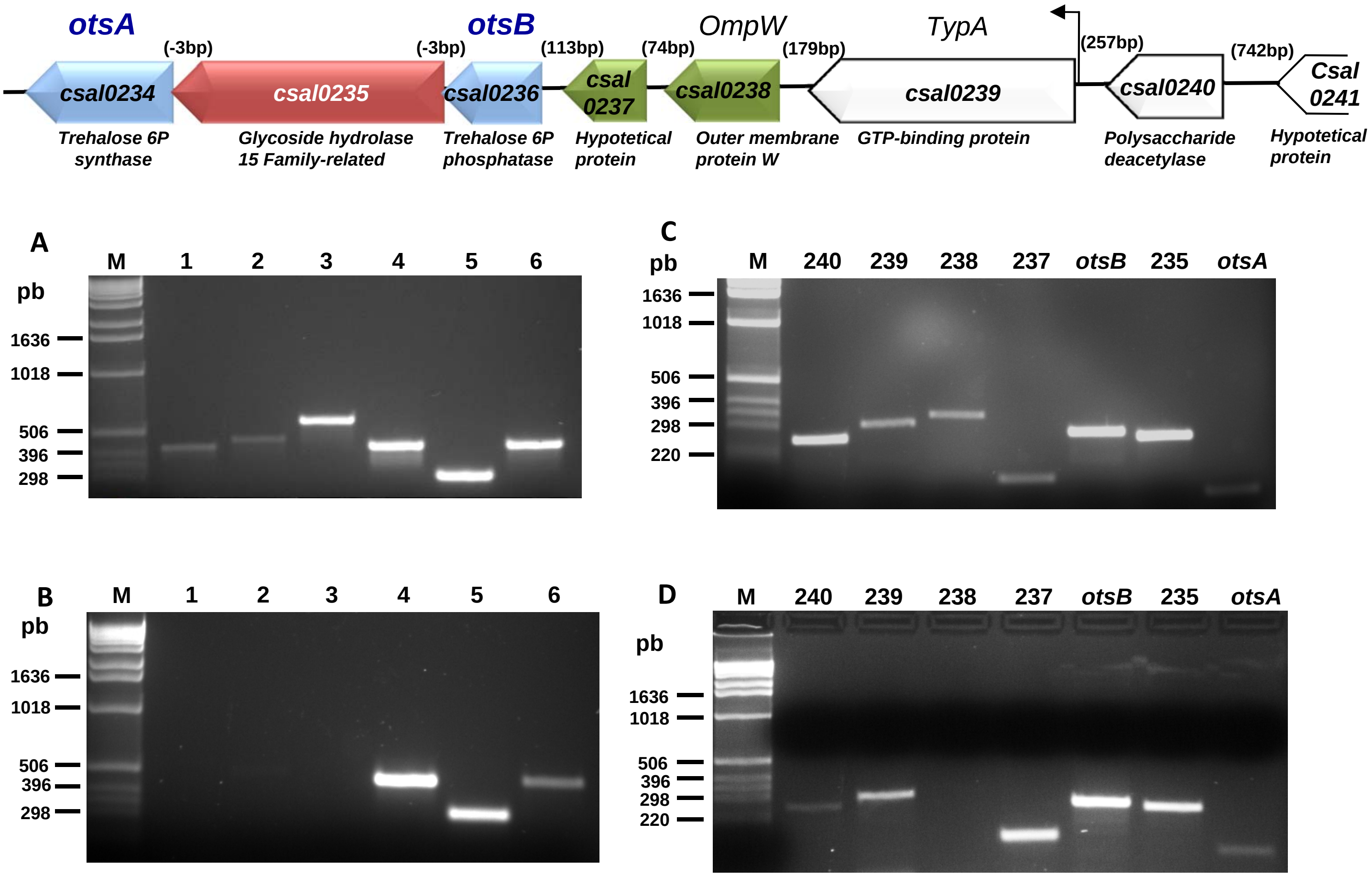

FIGURE S1. Genetic organization of *C. salexigens* trehalose synthesis gens *otsA* and *otsB*. Amplification of intergenic regions between *csal240* and *csal239* (lane 1), *csal239* and *csal238* (lane 2), *csal238* and *csal237* (lane3), *csal237* and *otsB* (lane 4), *otsB* and *csal235* (lane 5) and *csal235* and *otsA* (lane 6) genes by PCR using *C. salexigens* genomic DNA as template (A) or by RT-PCR (B). Intragenic regions of *csal240*, *csal239*, *csal238*, *csal237*, *otsB*, *csal235* and *otsA* genes were amplified by PCR using *C. salexigens* genomic DNA as template (C) or by RT-PCR (D), as a positive control of individual gene expression. cDNA was synthesized from RNA isolated form cultures of *C. salexigens* grown in M63 at 37ºC with 2.5 M NaCl. M, molecular weight marker (1 kb ladder, Invitrogen)
