# Supplementary material for: Role of Trehalose in Salinity and Temperature Tolerance in the Model Halophilic Bacterium Chromohalobacter salexigens
Source: PLoS One. 2012 Mar 20;7(3):e33587. doi: 10.1371/journal.pone.0033587 (PMC3308980; doi:10.1371/journal.pone.0033587)
Supplement: Table S1 — List of primers for RT-PCR assay. (PDF) [file pone.0033587.s002.pdf]

Supplementary Table 1. List of primers for RT-PCR assays

regions	gene	amplicon size (nt)	forward primer
**intragenic regions**			
csal240		259	CTCAGGTCCAGTGGCAAATTC
csal239		301	CTCCATGGTCTCCGGCAAG
csal238		330	CTATCGCTTCCACGACAAGTTC
csal237		150	GACAAGCAGCACGAAGTTCAG
csal236	*otsB*	271	CCGTCATTTCATGGCCGAATG
csal235		254	GCCAGTACTATCAGCGCAATAC
csal234	*otsA*	119	TCTTCCTGCACATTCCCTTTCC
**intergenic regions**			
csal240-csal239		433	CGTCTCTATGATTGGCTGCTC
csal239-csal238		470	GCACATCCGTCTGCGCAAG
csal238-csal237		581	CTATCGCTTCCACGACAAGTTC
csal237-csal236		431	GACAAGCAGCACGAAGTTCAG
csal236-csal235		297	CGCGACCTACTATCTTGAATCG
csal235-csal234		439	CCGAGAACGCCTTCAATATCTG

reverse primer
GAGCAGCCAATCATAGAGACG
CTTGCGCAGACGGATGTGC
CTGTCGATGTCCAGATACCAG
CTTGCCGTGGCTGTCGATG
CGATTCAAGATAGGTCGC
ACCGAGGTAGCCAATGTGATG
GCGATCGTTTTCGGTCTGAAAG
CTGGTCATTGGAGTCCATGATG
GAACTTGTCGTGGAAGCGATAG
CTGAACTTCGTGCTGCTTGTC
GGGTGCCATCGAAGTCCAG
GTATTGCGCTGATAGTACTGGC
CGTCGCGTAATCGATATTGTCG
